# Comprehensive Sexuality Education as a Longitudinal Predictor of LGBTQ Name-Calling and Perceived Willingness to Intervene in School

**DOI:** 10.1007/s10964-017-0638-z

**Published:** 2017-01-27

**Authors:** Laura Baams, Judith Semon Dubas, Marcel A. G. van Aken

**Affiliations:** 1 Developmental Psychology, Utrecht University, Heidelberglaan 1, Utrecht, 3584CS Netherlands; 2 Population Research Center, Human Development and Family Sciences, University of Texas at Austin, 23rd Street Stop G1800, Austin, TX 78712-1699 USA

**Keywords:** LGBTQ youth, School climate, Comprehensive sexuality education, Inclusive curricula, LGBTQ name-calling

## Abstract

Comprehensive sexuality education and sexuality education that is inclusive to lesbian, gay, bisexual, transgender, queer, and questioning (LGBTQ) youth is thought to educate and support youth in their social relations. Despite the obligation for Dutch schools to cover sexuality education in their curricula, including the topic of sexual diversity, the content that is covered varies widely across schools. With the current study, we present an overview of the content of sexuality education as reported by a sample of 601 Dutch adolescents (58.4% female youth) from six different high schools (e.g., public, Roman Catholic, protestant, anthroposophical; grades 10–12). Further, we examine whether the content or extensiveness of sexuality education at the beginning of the school year is related to a decrease in LGBTQ name-calling and an increase in the willingness to intervene when witnessing LGBTQ name-calling at the end of the school year. Adolescents completed three surveys, spaced four months apart. The results show that anatomy, STI prevention, and relationships are covered most often in sexuality education, with less attention to sexual diversity. Our longitudinal findings show that having a wide variety of topics covered in sexuality education—not just sexual diversity—was related to an increase in perceived willingness to intervene when witnessing LGBTQ name-calling by teachers or school staff, fellow students, and youth themselves (female youth). It also predicted a decrease in the occurrence of name-calling according to females. Our findings emphasize the importance of having comprehensive sexuality education in schools; it not only educates and empowers youth but also signals a safer school climate.

## Introduction

School should be a safe environment that promotes learning and development. However, for lesbian, gay, bisexual, transgender, queer, and questioning (LGBTQ) youth, school can be a dangerous place. Victimization for youth’s (presumed) sexual or gender identity is prevalent, and often even perpetrated by teachers and school personnel (Kosciw et al. 2016). Further, school personnel and fellow students rarely intervene when they witness such events (Kosciw et al. 2016). Having comprehensive sexuality education in school that is attentive to LGBTQ issues may signal a safer school climate with teachers and students who are aware of sexual diversity issues. For example, bringing attention to sexual diversity may encourage students to intervene when they witness LGBTQ name-calling. With the current study, we examine whether the specific topics covered (content) or the variety of topics covered (extensiveness) in sexuality education in Dutch high schools at the beginning of the school year is related to a decrease of LGBTQ name-calling, and an increase in the perceived willingness to intervene when witnessing LGBTQ name-calling at the end of the school year.

### Sexuality Education and Inclusive Curricula

Comprehensive sexuality education is described by the Sexuality Information and Education Council of the United States (SIECUS 2009) as “age-appropriate, medically accurate information on a broad set of topics related to sexuality including human development, relationships, decision making, abstinence, contraception, and disease prevention” (SIECUS 2009). *Inclusive* sexuality education or curricula also give attention to LGBTQ people and issues, and enable students and teachers to learn and talk about stereotypes and experiences of LGBTQ peers (Poteat et al. 2014). Research has confirmed that, in general, inclusive curricula are related to a safer school climate for LGBTQ students (e.g., Greytak et al. 2013; Kosciw et al. 2013; Snapp et al. 2015; Toomey et al. 2012).

While the majority of curricula in high schools are not inclusive to LGBTQ issues, the extensiveness of sexuality education, or comprehensive sexuality education may serve a similar purpose. Research has shown that having comprehensive sexuality education in school, compared to abstinence only programs, decreases rates of teen pregnancy (Kohler et al. 2008) and increases condom and contraceptive use (Kirby et al. 2007). Similarly, countries with comprehensive sexuality education programs, such as the Netherlands, have better sexual health outcomes such as lower rates of pregnancy, births, and abortions (Weaver et al. 2005).

In addition to learning about potential risks of sexual behavior, school is also a context in which youth can learn about diversity and positive ways of interacting with youth who may be different from themselves. As such, the school’s curricula may help to improve school climate and conversations around sexual diversity. Curricula may provide students with an understanding for youth who are different from themselves, and curricula could model positive behavior toward those peers (Slaatten et al. 2015). Style describes these functions of curricula as being a “mirror” for the person, and a “window” into the lives of others (Style 1996). Thus, youth can see themselves *and* learn about others through their curricula.

An area where attention was first brought to inclusiveness of curricula was research on ethnic studies (Snapp and Russell, & the Crossroads Collaborative 2017). In a review of the literature, Sleeter (2011) found that having ethnic studies in school’s curriculum had positive effects for student’s self-worth and empathy as well as their attitudes toward ethnic minorities (e.g., Sleeter 2011). Building on this work, scholars have suggested that curriculum that is inclusive to LGBTQ issues may also diminish stereotypes and biases around gender and sexual orientation, and thus create a safer school climate for LGBTQ students (Goodenow et al. 2006; Kosciw et al. 2013; Snapp et al. 2015; Toomey et al. 2012). One such study found that, in schools with LGBTQ-inclusive curricula, students reported feeling safer and reported lower levels of bullying compared to students in schools that did not have such inclusive curricula (Snapp et al. 2015). Because the content of comprehensive sexuality education and inclusive curricula differs across schools, states, and countries (Inspectie van het Onderwijs 2016; Pound et al. 2016), it is unclear what the effective components of curricula would be in improving school climate.

### Experiencing and Witnessing LGBTQ Name-Calling

Many LGBTQ youth face discrimination in the form of verbal and physical harassment and exclusion. Of U.S. LGBTQ students, 70.8 and 54.5% report being verbally harassed because of their sexual orientation or their gender expression in the past school year, and 27.0 and 20.37% of LGBTQ students report being physically harassed because of their sexual orientation or gender expression (Kosciw et al. 2016). While the prevalence of victimization in the U.S. is higher, in the Netherlands LGB youth also report high rates of victimization. In a Dutch LGB sample, 40% reported having had a negative experience related to their sexuality in the last 12 months, 15% of these youth reported that the perpetrator of the victimization was a peer in school, 6% reported that the perpetrator was school personnel (Kuyper 2015). Further, terms like “gay” or “dyke” are regularly used as derogatory terms in Dutch high schools, 88% of a sample of Dutch high school students reported hearing such terms sometimes to (very) often (Kuyper 2015). The prevalence of harassment and victimization of LGBTQ youth has been documented in multiple national and convenience samples and across various countries (Collier et al. 2013). Consequences of these negative experiences include higher rates of depression and suicidality (Collier et al. 2013; Russell and Fish 2016), especially during adolescence (Marshal et al. 2013).

Name-calling in school, without the intervention of a teacher, validates the messages that LGBTQ youth receive and leads to a social environment in which such behaviors are encouraged and accepted. Two longitudinal studies showed that in peer groups in which gay-related name-calling was common, youth increased their own use of gay-related name-calling over time (Birkett and Espelage 2015; Poteat 2007). In contrast, schools in which teachers intervene when they witness name-calling may have a lower occurrence of name-calling. For example, a study among ninth grade students in Norway (Slaatten et al. 2015) showed that observing teachers intervene when students engaged in gay-related name-calling was related to a lower occurrence of gay-related name-calling. Similarly, a study on students’ willingness to intervene when witnessing anti-transgender harassment showed that hearing transphobic language in school predicted a lower likelihood to intervene, while witnessing other students intervene predicted a higher likelihood to intervene (Wernick et al. 2014). Thus, observing name-calling in the peer group is important for the improvement or sustainment of youth’s attitudes and behaviors related to gender and sexual diversity. However, having teachers who condemn gay-related name-calling and intervene when they witness it may help to create a school climate in which such behaviors are unacceptable (Slaatten et al. 2015). Despite attention for bystanders in the bullying literature (Salmivalli et al. 2011), there is little research on factors that may encourage students and school personnel to intervene when they witness LGBTQ name-calling.

### Comprehensive Sexuality Education and School Climate in The Netherlands

Although many consider the Netherlands to be a liberal country that is accepting of gender and sexual diversity (Kuyper et al. 2013), Dutch LGBTQ youth experience victimization and bullying for their sexual and gender identity (Kuyper 2015). Terms like “gay” or “dyke” are regularly used as derogatory terms in Dutch high schools, and similar to findings elsewhere, Dutch LGBTQ youth report poorer mental and physical health compared to their heterosexual peers (Kuyper 2015).

Starting in 2012, all secondary schools in the Netherlands are obligated to cover sexual diversity in their curriculum. The goals of this curriculum are phrased as follows: “the student learns about similarities, differences and changes in culture and world-view in the Netherlands; learns to link their own and others’ ways of life to this; learns the meaning for society to respect each other’s attitudes and ways of life; and learns to respectfully handle sexuality and diversity in society, including sexual diversity” (Kamerstuk Staten Generaal 2012). A recent report by the Dutch School Inspection indicated that schools differ in their efforts to accomplish these goals. About 14% of principals and 29% of teachers reported that sexual diversity was not covered in their curriculum (Inspectie van het Onderwijs 2016). In schools in which sexual diversity *was* covered, this was usually in both middle and high school (83%), and most often in the biology, sociology, or religion classes. For schools that did not cover sexual diversity, this was attributed to a perceived lack of expertise or a perceived lack of support among personnel (Inspectie van het Onderwijs 2016). In sum, although the Netherlands has pragmatic and sex positive government policies, especially compared to other Western countries (Weaver et al. 2005), it is currently unknown whether the extensiveness of sexuality education or the specific topics in sexuality education may help to create a safer school climate.

## Current Study

With the current study, we present an overview of the content of sexuality education as reported by a sample of 601 Dutch adolescents from six different high schools (grades 10–12). Further, we examine whether the specific content of sexuality education or the extensiveness of sexuality education is related to an improvement of school climate over time. Based on empirical work (see Snapp et al. 2015 for a recent overview), we hypothesize that extensive sexuality education and, specifically, the topic of sexual orientation and gender predicts a decrease in the occurrence of LGBTQ name-calling over time, and an increase in perceived willingness to intervene by fellow students, school personnel, and youth themselves.

Further, research among LGBTQ youth shows that males experience more name-calling in comparison to females (van Beusekom et al. 2016), and that females are more likely to intervene when homophobic behavior occurs (Poteat and Vecho 2016). However, despite these mean-level differences, we do not have specific hypotheses on sex differences of associations between sexuality education and school climate, therefore we explore a multi-group model for male and female youth to examine possible differences.

## Method

### Procedure and Sample Characteristics

Data were collected in six high schools in the Netherlands, as part of a larger three-wave longitudinal research project (grades 10 to 12). These schools were selected to represent different urban, suburban, and rural areas in the Netherlands varying in size and denomination. The first measurement wave took place in the fall of 2014, two subsequent measurement waves took place after 4 and 8 months. In the current study, we used the first and third measurement wave.

Permission for this study and its protocol was granted by the ethics board of the Faculty of Social and Behavioural Sciences of Utrecht University, the Netherlands. A total of 622 students and their parents were informed of the study. If the student’s parents did not contest their participation *and* the student consented to participating, they could participate in the study (9 out of 622 students did not participate). Several gift certificates and electronic tablets were raffled among the participants. Two student-assistants introduced the topics, emphasized the confidentiality of the study and students’ voluntary participation, and remained present at all times to answer any questions. After each measurement wave, all participants received contact information concerning organizations and resources that provide information on gender and sexuality.

To be included in the longitudinal sample, participants had to complete a question on their biological sex, but they were free to skip all other questions in the survey. This resulted in a longitudinal sample of 601 adolescents who participated in at least one of the measurement waves (ages 14–18; *M* = 15.8, SD = 0.8; 250 male youth and 351 female youth). Most participants reported a Dutch cultural background (95.1%), were enrolled at a pre-university level (52.0%), and reported being heterosexual when asked about their sexual identity (96.5%).

There was significant attrition between the first and third measurement wave (28%). However, differences between the included sample and those that dropped out were limited (Nagelkerke R Square = .09): Participants who dropped out were older (*B* = −.42) and reported a higher frequency of the topic “STI prevention” in their sexuality education (*B* = −.30). We used full information maximum likelihood (FIML) to handle missing data, this resulted in a final sample size of 577 for the multi-group models (245 male youth; 332 female youth). Results of a complete case analysis did not differ from the analyses using FIML.

### Measures

#### Sexuality education

We used three variables to assess content and extensiveness of sexuality education. The first pertains to the specific topics that are covered in sexuality education (content). For this variable, students were presented with five different topics: Sexual orientation and gender, resources, STI prevention, relationships, and anatomy (adapted from Gowen and Winges-Yanez 2014) and asked the following question “Are the following issues ever mentioned in class or a lesson?”All topics were presented with examples: Sexual orientation and gender: “such as sexual orientation, LGBT people, gender expression,” resources: “such as folders, online resources, local resources,” STI prevention: “such as STI preventions, safer sex, condoms,” relationships: “such as communication, healthy relationships, dating violence,” and finally, anatomy: “such as different body parts, diversity in external appearance, body acceptance” (see Table 1 for descriptive statistics for the overall sample and by school). Participants reported the frequency with which these five topics were covered in their curriculum on a 5-point Likert scale (0 = *never* to 4 = *a lot*). We also constructed a second variable: Extensiveness of sexuality education. This variable represents the extensiveness of sexuality education by summing the frequency of the five previous items about topics (*M* = 11.97, SD = 4.24). Thus, a higher score indicated that a student had more topics and/or a higher frequency of these topics in their sexuality education. The coverage of the five topics was strongly correlated (α = .84). Last, we constructed a third variable: Number of topics covered in sexuality education. To create this variable we recoded answers 2–4, as 1 (*present*) and kept the category 0 (*never*). Then, we summed these five dichotomous variables to create a sum score of topics that were covered in sexuality education.

**Table 1:** Percentages and means of the frequency with which topics are covered in sexuality education for the overall sample and by school

						Overall^a^ *N* = 601	School 1 *n* = 155	School 2 *n* = 66	School 3 *n* = 88	School 4 *n* = 72	School 5 *n* = 75	School 6 *n* = 145
	0. Never	1.	2.	3.	4. A lot	*M* (SD)	*M* (SD)	*M* (SD)	*M* (SD)	*M* (SD)	*M* (SD)	*M* (SD)
Sexual orientation and gender	27.1	31.7	28.1	11.2	1.8	2.29 (1.04)	1.84 (0.95)	2.33 (0.89)	3.02 (1.08)	2.49 (0.91)	2.49 (1.09)	2.25 (1.02)
Resources	36.1	31.3	22.6	9.0	1.0	2.08 (1.02)	1.67 (0.92)	2.03 (0.89)	2.74 (1.16)	2.17 (0.97)	2.32 (1.03)	2.09 (0.95)
STI prevention	24.2	26.5	28.5	18.2	2.6	2.48 (1.12)	2.12 (1.14)	2.34 (0.96)	3.02 (1.05)	2.65 (0.97)	3.03 (1.13)	2.37 (1.10)
Relationships	21.6	26.7	35.3	14.0	2.4	2.49 (1.05)	2.31 (1.13)	2.78 (1.02)	2.81 (1.00)	2.38 (0.97)	2.73 (0.98)	2.34 (1.02)
Anatomy	22.1	21.5	32.1	19.9	4.4	2.63 (1.16)	2.46 (1.21)	2.64 (1.07)	3.11 (1.09)	2.54 (1.09)	2.75 (1.17)	2.59 (1.16)

^a^ Participants were presented with five different topics and asked how often these were ever discussed in the classroom or in a lesson. Item response options were on a 5-point Likert scale (0 = *never* to 4 = *a lot*). Adapted from Gowen and Winges-Yanez (2014)

#### The occurrence of LGBTQ name-calling and perceived willingness to intervene

The occurrence of LGBTQ name-calling (adapted from Wernick et al. 2014) was assessed with the following item: “Sometimes people use phrases such as “gay” or “fag” that are derogatory toward gay, lesbian, and bisexual people. How often do you hear phrases like the above in school?” Participants reported the frequency of these occurences on a 5-point Likert scale (1 = *never* to 5 = *frequently*). Further, three items were used to assess perceived willingness to intervene when witnessing LGBTQ name-calling (adapted from Wernick et al. 2014). To assess the perceived willingness to intervene by teachers or other school personnel and fellow students two items were used: “When present, how often do [teachers or other school staff] [other students] intervene when phrases like “gay” or “fag” are made?” Participants reported the frequency of these occurences on a 5-point Likert scale (1 = *never* to 5 = *frequently*). Last, one item was used to assess the likelihood with which youth themselves would intervene: “How likely are you to intervene if you saw or heard harassment based on sexual orientation.” Participants reported their likelihood to intervene on a 5-point Likert scale (1 = *not likely* to 5 = *likely*). Correlations between the recoded item on LGBTQ name-calling and the three items on perceived willingness to intervene range from *r* = .10 to .21.

#### Demographics

Several demographics were included in the current study. Biological sex was assessed with the following item: “In your passport or identification card, does it state that you are male or female?” Education level was assessed according to the current Dutch education levels to which most students are assigned in seventh grade. Cultural background was assessed with the following item: “How would you describe your cultural background.” Youth could respond to the following options: Dutch, Suriname, Antillean or Aruban, Turkish, Moroccan, Surinam Dutch, Antillean or Aruban Dutch, Turkish Dutch, Moroccan Dutch, or Other, namely… A single item assessed sexual identity: “When you think about your sexual identity, do you think of yourself as…” Youth could respond with Lesbian, Gay, Bisexual, Queer, Straight, or Other.

### Analyses

To examine whether the extensiveness and content of sexuality education at measurement wave 1 predicted perceived willingness to intervene when witnessing LGBTQ name-calling and the occurrence of LGBTQ name-calling at measurement wave 3, one model for the *content* of sexuality education and two models for the *extensiveness* of sexuality education were estimated in Mplus version 7.3.1 (Muthén and Muthén 1998–2012) by using maximum likelihood estimation with robust standard errors (MLR). Because we included multiple predictors and covariates into the models, the number of schools was too small to run multilevel analyses. However, intraclass correlations were low (0.016–0.068). We present the results for the full sample of *N* = 577. In all analyses we control for school climate at wave 1, education level, and age.

## Results

### Content of Sexuality Education

Participants reported the frequency of several topics being covered in their sexuality education (See Table 1 for descriptive statistics for the overall sample and by school). Overall, the results show that a large percentage of students reported that topics are “never” covered in their sexuality education. Especially, resources and sexual orientation and gender are topics that are rarely covered (36.1 and 27.1% of students report that resources and sexual orientation and gender, respectively, were “never” covered). However, STI prevention, relationships, and anatomy are covered most often in sexuality education. With four paired sample t-tests, we examined whether sexual orientation and gender was covered more or less often than the other four topics. In general, sexual orientation and gender was covered more often than resources (*t*(497) = 5.20, *p* < .001), and less often than STI prevention (*t*(497) = −4.07, *p* < .001), relationships (*t*(497) = −4.11, *p* < .001), and anatomy (*t*(496) = −6.52, *p* < .001).

### Gender Differences in School Climate

Table 2 presents descriptive statistics of school climate for the overall sample and by school. With a MANOVA, male and female youth were found to differ in their assessment of school climate (Pillai’s trace = .13, *F*(4, 363) = 13.38, *p* < .001, *η* ^2^_p_ = .129; see Table 2). Univariate analyses show that females reported a higher tendency to intervene when witnessing LGBTQ name-calling than males (*p*s < .05). Females also reported a higher perceived likelihood that their fellow students would intervene (*p* < .05). Further, females reported a lower occurrence of LGBTQ name-calling than males (*p* < .05).

**Table 2:** Descriptive statistics of school climate at wave 3 for male and female youth and by school

	Male	Female		School 1	School 2	School 3	School 4	School 5	School 6
	*M* (SD)	*M* (SD)	% never^e^	*M* (SD)	*M* (SD)	*M* (SD)	*M* (SD)	*M* (SD)	*M* (SD)
*Perceived willingness to intervene by:*									
Teachers or school personnel^a^	2.45 (1.17)	2.46 (1.14)	22.4	2.31 (1.09)	2.51 (1.07)	2.82 (1.29)	2.67 (0.58)	2.63 (0.92)	2.40 (1.20)
Fellow students^a^	1.63 (0.98) ^c^	1.93 (1.01)	50.5	1.79 (0.95)	1.75 (1.04)	2.04 (1.09)	1.67 (0.58)	1.25 (0.46)	1.79 (1.04)
Youth themselves^b^	2.63 (1.10) ^c^	3.07 (1.02)	11.1	2.96 (1.05)	2.79 (1.13)	2.91 (0.99)	3.00 (1.73)	3.25 (1.17)	2.83 (1.10)
Occurrence of LGBTQ name-calling^d^	3.46 (1.36) ^c^	2.64 (1.15)	–	3.08 (1.26)	2.77 (1.31)	2.40 (1.16)	4.33 (1.16)	3.38 (1.41)	3.17 (1.33)

^a^ Participants reported the frequency of these occurences on a 5-point Likert scale (1 = *never* to 5 = *frequently*)

^b^ Participants reported their likelihood to intervene on a 5-point Likert scale (1 = *not likely* to 5 = *likely*)

^c^ Univariate difference between male and female youth

^d^ Participants reported the frequency of these occurences on a 5-point Likert scale (1 = *never* to 5 = *frequently*)

^e^ Percentage of participants who reported that they themselves, their teachers, or their fellow students “never” intervened when witnessing LGBTQ name-calling

### Sexuality Education and School Climate

#### Content of sexuality education

To assess whether the different aspects of sexuality education were related to a *change* in school climate over time, we entered the five items on sexuality education topics as predictors of four outcomes of school climate, controlling for school climate at measurement wave 1, education level, and age. The multi-group model for male and female youth showed a better fit (RMSEA = .00, CI[.00, .05], CFI = 1.00, TLI = 1.01) than the constrained model (RMSEA = .03, CI[.00, .05], CFI = .96, TLI = .93). Therefore, the results are reported separately for male and female youth (Tables 3 and 4, respectively).

**Table 3:** The content of sexuality education predicting school climate for male youth

	Willingness to intervene when witness LGBTQ name-calling by:							
	Teachers or school personnel		Fellow students		Youth themselves		Occurrence of LGBTQ name-calling	
	*β* (SE)	*p*	*β* (SE)	*p*	*β* (SE)	*p*	*β* (SE)	*p*
Education level	.10 (.09)	.278	.38 (.09)	<.001	.09 (.08)	.270	.11 (.09)	.231
Age	.02 (.08)	.850	.13 (.09)	.160	.04 (.09)	.683	.17 (.07)	.012
Controlling for wave 1	.40 (.08)	<.001	.04 (.09)	.626	.52 (.07)	<.001	.49 (.07)	<.001
*Content of sexuality education*								
Sexual orientation and gender	−.13 (.10)	.196	.11 (.11)	.323	−.02 (.08)	.842	.06 (.09)	.539
Resources	.07 (.11)	.521	.02 (.13)	.906	−.07 (.10)	.483	−.05 (.12)	.652
STI prevention	.06 (.13)	.629	.00 (.15)	.987	.30 (.11)	.007	.12 (.13)	.367
Relationships	.23 (.10)	.018	−.12 (.11)	.244	−.00 (.08)	.966	−.03 (.10)	.785
Anatomy	.03 (.10)	.747	.04 (.13)	.775	−.04 (.11)	.694	.05 (.11)	.694

**Table 4:** The content of sexuality education predicting school climate for female youth

	Willingness to intervene when witness LGBTQ name-calling by:							
	Teachers or school personnel		Fellow students		Youth themselves		Occurrence of LGBTQ name-calling	
	*β* (SE)	*p*	*β* (SE)	*p*	*β* (SE)	*p*	*β* (SE)	*p*
Education level	.00 (.09)	.971	.06 (.07)	.451	.01 (.10)	.906	−.08 (.08)	.309
Age	.06 (.08)	.418	.02 (.07)	.792	−.03 (.07)	.663	.11 (.06)	.062
Controlling for wave 1	.31 (.07)	<.001	.28 (.07)	<.001	.43 (.07)	<.001	.55 (.06)	<.001
*Content of sexuality education*								
Sexual orientation and gender	−.05 (.09)	.603	−.04 (.10)	.674	.08 (.11)	.420	−.08 (.08)	.332
Resources	.13 (.09)	.164	.17 (.09)	.063	−.09 (.08)	.267	.04 (.08)	.644
STI prevention	.18 (.10)	.065	−.01 (.11)	.947	.05 (.11)	.672	−.12 (.08)	.136
Relationships	.09 (.09)	.329	−.02 (.08)	.853	.14 (.09)	.107	−.06 (.07)	.354
Anatomy	.04 (.09)	.686	.21 (.09)	.014	−.04 (.10)	.696	.05 (.07)	.521

Overall, the five different topic of sexuality education did not predict changes in the perceived willingness to intervene by teachers or school personnel, fellow students, and youth themselves when witnessing LGBTQ name-calling. Nor did they predict a decrease in the occurrence of LGBTQ name-calling. However, three findings stood out: For males, having curriculum in which STI prevention is frequently covered was related to an increase in the willingness to intervene when witnessing LGBTQ name-calling (*β* = .30, *p* = .007). Further, for males, having curriculum in which relationships are frequently covered was related to an increase in perceived willingness to intervene by teachers or school personnel when witnessing LGBTQ name-calling (*β* = .23, *p* = .018). Finally, for females, having curriculum in which anatomy is frequently covered was related to an increase in perceived willingness to intervene by fellow students when witnessing LGBTQ name-calling as reported (*β* = .21, *p* = .014).

#### Extensiveness of sexuality education

To examine whether the extensiveness of sexuality education predicted change in the occurrence of LGBTQ name-calling and perceived likelihood of intervening, we used two measures of “extensiveness,” 1) we conducted a regression analysis including a sum score of the five topics and their frequencies (ranging from 0 to 20), and 2) we conducted a regression analysis including a sum score of the dichotomized items assessing the number of topics present in sexuality education (ranging from 0 to 5).

First, two multi-group models were estimated using the sum score of the five topics and their frequencies. The unconstrained multi-group model for male and female youth (RMSEA = .00, CI[.00, .04], CFI = 1.00, TLI = 1.02) showed a better fit than the constrained model (RMSEA = .03, CI[.00, .06], CFI = .97, TLI = .94). Therefore, the results are reported separately for male and female youth (Table 5). For both males and females, having more extensive sexuality education at the beginning of the school year was related to an increase over time in perceived willingness to intervene by teachers or school personnel when witnessing LGBTQ name-calling (Males: *β* = .22, *p* < .001; Females: *β* = .30, *p* < .001). More extensive sexuality education was related to an increase over time in perceived willingness to intervene by fellow students, but only among females (*β* = .26, *p* < .001), not among males (*β* = .02, *p* = .719). Finally, more extensive sexuality education was related to an increase over time in the willingness to intervene by themselves, but only among males (*β* = .15, *p* = .031), not among females (*β* = .12, *p* = .106). Concerning the occurrence of LGBTQ name-calling, the results show that having more extensive sexuality education was related to a decrease in the occurrence of LGBTQ name-calling, but only among females (*β* = −.14, *p* = .021) and not among males (*β* = .11, *p* = .130).

**Table 5:** The extensiveness of sexuality education predicting school climate for male and female youth

	Perceived willingness to intervene when witnessing LGBTQ name-calling by:							
	Teachers or school personnel		Fellow students		Youth themselves		Occurrence of LGBTQ name-calling	
	*β* (SE)	*p*	*β* (SE)	*p*	*β* (SE)	*p*	*β* (SE)	*p*
*Male*								
Education level	.09 (.09)	.321	.39 (.09)	<.001	.07 (.09)	.390	.11 (.09)	.222
Age	.04 (.08)	.648	.11 (.09)	.216	.05 (.09)	.618	.16 (.07)	.014
Controlling for wave 1	.39 (.08)	<.001	.04 (.09)	.635	.51 (.07)	<.001	.50 (.07)	<.001
Extensiveness of sexuality education	.22 (.06)	<.001	.02 (.07)	.719	.15 (.07)	.031	.11 (.07)	.130
*Female*								
Education level	−.00 (.09)	.983	.07 (.07)	.372	.01 (.10)	.934	−.07 (.08)	.376
Age	.06 (.08)	.429	.04 (.07)	.604	−.04 (.07)	.588	.12 (.06)	.044
Controlling for wave 1	.30 (.07)	<.001	.27 (.07)	<.001	.42 (.07)	<.001	.56 (.06)	<.001
Extensiveness of sexuality education	.30 (.08)	<.001	.26 (.07)	<.001	.12 (.07)	.106	−.14 (.06)	.021

Second, two multi-group models were estimated using the sum score of the dichotomized items assessing the presence of topics in curriculum. The unconstrained multi-group model for male and female youth (RMSEA = .00, CI[.00, .05], CFI = 1.00, TLI = 1.00) showed a better fit than the constrained model (RMSEA = .03, CI[.00, .05], CFI = .97, TLI = .96). Therefore, the results are reported separately for male and female youth (Table 6). For both males and females, having a higher number of topics covered in sexuality education at the beginning of the school year was related to an increase over time in perceived willingness to intervene by teachers or school personnel when witnessing LGBTQ name-calling (Males: *β* = .19, *p* = .005; Females: *β* = .16, *p* = .020). A higher number of topics in sexuality education was also related to an increase over time in perceived willingness to intervene by fellow students, but only among females (*β* = .14, *p* = .030), and not among males (*β* = .01, *p* = .922). Finally, the number of topics in sexuality education was not related to a change in the willingness to intervene for students themselves (Males: *β* = .14, *p* = .055; Females: *β* = .04, *p* = .578). Concerning the occurrence of LGBTQ name-calling, the results show that the number of topics in sexuality education was also not related to a change over time in the occurrence of LGBTQ name-calling (Males: *β* = .04, *p* = .648; Females: *β* = −.10, *p* = .097).

**Table 6:** The number of topics present in sexuality education predicting school climate for male and female youth

	Perceived willingness to intervene when witnessing LGBTQ name-calling by:							
	Teachers or school personnel		Fellow students		Youth themselves		Occurrence of LGBTQ name-calling	
	*β* (SE)	*p*	*β* (SE)	*p*	*β* (SE)	*p*	*β* (SE)	*p*
*Male*								
Education level	.10 (.09)	.306	.39 (.09)	<.001	.07 (.08)	.396	.12 (.10)	.196
Age	.04 (.08)	.661	.11 (.10)	.241	.05 (.10)	.615	.15 (.07)	.020
Controlling for wave 1	.38 (.08)	<.001	.05 (.09)	.559	.52 (.07)	<.001	.50 (.07)	<.001
Number of topics	.19 (.07)	.005	.01 (.08)	.922	.14 (.08)	.055	.04 (.08)	.648
*Female*								
Education level	−.02 (.10)	.841	.05 (.08)	.501	−.00 (.10)	.995	−.06 (.08)	.411
Age	.04 (.08)	.619	.02 (.07)	.806	−.06 (.08)	.467	.12 (.06)	.051
Controlling for wave 1	.29 (.07)	<.001	.27 (.07)	<.001	.42 (.07)	<.001	.56 (.06)	<.001
Number of topics	.16 (.07)	.020	.14 (.06)	.030	.04 (.08)	.578	−.10 (.06)	.097

### Sensitivity Analyses

With a MANOVA, we found that schools differed in the frequency with which topics were covered in their sexuality education at measurement wave 1 (Pillai’s trace = .22, *F*(25, 2455) = 4.52, *p* < .001, *η* ^2^_p_ = .044). Posthoc analyses revealed that students in School 1 reported the lowest frequency of topics covered in their sexuality education compared to all other schools. Further, schools differed in student’s perception of school climate across schools at measurement wave 1 (Pillai’s trace = .14, *F*(20, 2036) = 3.66, *p* < .001, *η* ^2^_p_ = .035). Posthoc analyses show that students in School 5 reported the highest level of LGBTQ name-calling compared to Schools 1 to 4 (*p* < .05). There were no consistent differences in perceived willingness to intervene by teachers or school personnel, fellow students, or youth themselves.

Because our findings showed some mean-level differences between schools, we examined whether adding the school identifier as dummies (School 1 was the reference category) would alter the results. The models on content (five different topics) did not converge due to non-variance in several schools. For the two models on the sum scores (extensiveness), the analyses yielded similar results.

## Discussion

Previous research has suggested that a curriculum that is inclusive to sexual diversity and LGBTQ issues would improve school climate (e.g., Greytak et al. 2013; Kosciw et al. 2013; Snapp et al. 2015; Toomey et al. 2012). Whether comprehensive sexuality education, and the topic of sexual orientation and gender specifically, would also be related to a change in school climate had not been researched before. In the current study, we used a longitudinal design to examine the content of sexuality education in six Dutch high schools at the beginning of the school year, and tested whether individual topics or the extensiveness of sexuality education would be related to an increase in the willingness to intervene when witnessing LGBTQ name-calling and a decrease in LGBTQ name-calling across the school year.

The findings of the current study consist of three parts. First, concerning the content of sexuality education, our findings show that sexual orientation and gender is rarely covered in sexuality education. Sexual orientation and gender as a topic is covered more often than resources, but less often than STI prevention, relationships, and anatomy. Although the findings in the current sample of high schools may not be generalizable to other schools, they do show that, despite the importance of inclusive sexuality education (Snapp et al. 2015), and the obligation to cover sexual diversity in the Dutch school system, sexual orientation, and gender was not regularly included in schools’ sexuality education programs, as perceived by students.

Second, we examined whether the content of sexuality education was related to changes in school climate across the school year. We expected that curricula on sexual orientation and gender would be related to a change in school climate. However, the findings only showed that having STI prevention (for males), relationships (for males), and anatomy (for females) in curricula were related to a change in school climate. The lack of findings for the topic of sexual orientation and gender may be explained by the low frequency of coverage. There may be a “critical mass” of inclusive and supportive curricula (Snapp et al. 2015, p. 590) that is required to effectively improve the school climate. The schools in the current sample may not have reached this “critical mass,” indicated by their low coverage of sexual orientation and gender in the curriculum. Further, we did not assess what the messages were that youth received about sexual orientation and gender. These messages may not be supportive or affirming, or not taught by supportive teachers. Corroborating what was found in a recent report by the Dutch School Inspection, the schools in our sample often did not cover sexual diversity in their curricula, and this may be because schools lack programs to teach about sexual diversity, and teachers feel inadequate to teach about sexual diversity (Inspectie van het Onderwijs 2016). It is also possible that inclusive curricula improves school climate in ways that were not assessed in the current study (they may create a sense of understanding of diversity and increase acceptance or negate negative messages that youth experience; Snapp et al. 2015). As noted above, a more comprehensive assessment of curricula is needed to draw conclusions about content effects.

Third, we examined whether the extensiveness of sexuality education was related to an improvement in school climate. Confirming our hypotheses, we found that having more extensive sexuality education, and having more topics covered, was related to an increase in the willingness to intervene when witnessing LGBTQ name-calling by teachers and school personnel, according to both males and females. For female youth, we also found that the extensiveness of sexuality education was related to the perceived willingness to intervene by fellow students. For male youth, we found this relation for the willingness to intervene themselves. Finally, we found that more extensive sexuality education was related to a reduction in name-calling according to females, but not males.

Although we did not assess when the students had received sexuality education, even when we control for age of the adolescents the findings show that having more extensive sexuality education was related to a change of school climate across the school year. In other words, extensive sexuality education may facilitate or speed-up the development of acceptance of gender and sexual diversity in schools. Although we should interpret these correlational findings with some caution—causal mechanisms cannot be inferred—the findings do expand existing literature (Greytak et al. 2013; Kosciw et al. 2013; Snapp et al. 2015; Toomey et al. 2012) and indicate that comprehensive sexuality education may help to raise awareness of gender and sexual diversity.

### Differences Between Male and Female Youth

Because males and female differ in terms of experienced LGBTQ name-calling (van Beusekom et al. 2016) and the likelihood of intervening when homophobic behavior occurs (Poteat and Vecho 2016), we examined sex differences in the associations between sexuality education and school climate. Similar to previous studies, we found that females tend to show more defender behavior against LGBTQ name-calling than males (Poteat and Vecho 2016), and males tend to witness more LGBTQ name-calling than females (Slaatten et al. 2015), and males are also less likely to intervene when witnessing harassment (Wernick et al. 2014). We also found that sexuality education was differentially related to changes in school climate for male and female youth. This may indicate that male and female youth require different interventions to encourage accepting attitudes and behavior toward sexual and gender minority peers. For example, it has been suggested that, to become more accepting of sexual diversity, boys would require a change in masculinity norms (Poteat et al. 2011). Unfortunately, there is very little research that theorizes on how girls’ acceptance of gender and sexual diversity may be increased, and therefore we stress the importance of considering gender in research on (preventive) interventions.

### Strengths, Limitations, and Suggestions for Future Research

There are several strengths of the current study that extend or improve existing research. First, the present design included measures of the content and extensiveness, not merely existence, of sexuality education. Further, we assessed school climate with four different measures, assessing both the occurrence of LGBTQ name-calling as well as the perceived likelihood of persons to intervene when they witness LGBTQ name-calling. Because these measures infer the perception that fellow students and teachers or other school personnel would intervene when they witness LGBTQ name-calling, we may circumvent some of the social desirability issues that arise when we would *only* ask about student’s own likelihood to intervene. Second, the current study is one of the first to use a longitudinal design. This enables the assessment of changes in school climate across the school year.

The current study also has several limitations. The first limitation pertains to our assessment of sexuality education and school climate. Our assessment of sexuality education taps into five areas of content that may be covered in lessons and class. We did not assess the timing and tone of the curricula, or the detailed content of lessons that were taught in school. Further, we only assessed students’ perceptions of the topics and whether these were covered. Some students may not accurately remember or report the frequency with which these topics were covered in their curricula. Considering the wide range of sexuality education programs available, it is also possible that we assessed a cumulative effect of sexuality education, possibly across a period of several years. For future research on inclusive curricula, it would be important to assess the timing and content of curricula, the tone of the messages they send, and the supportiveness of teachers and school personnel in teaching these curricula. This way, we would be able to investigate the effective components and didactic strategies that increase acceptance of diversity. Further, our assessment of school climate focused mainly on perceived defender behaviors. This raises some concerns about selective perception: Some youth may differ in their interpretation or observation of defender behaviors. Moreover, because we only included information from the students themselves, shared-method variance should be considered. Including multiple informants such as students *and* teachers would improve the assessment of defender behaviors.

Our second limitation pertains to the sample characteristics of the current study. We included a sample of six high schools in the Netherlands, and most of the students reported a heterosexual sexual identity—similar to other large longitudinal studies in the Netherlands (e.g., Baams et al. 2014; Reitz et al. 2015). Although using a general school population sample enables the generalization of findings to non-LGBTQ students, it did not enable us to examine the different perceptions of curricula and discrimination that LGBTQ students may have (e.g., Snapp et al. 2015). Unfortunately, the limited number of schools also did not allow for multilevel testing. Therefore, we cannot examine potentially important school-level factors that may account for some of our findings, such as policies and school programs related to bullying. Further, because our sample was homogeneous in cultural background, the findings of the current study may not directly generalize to the general adolescent population.

Third, it is important to note that the current study has a correlational longitudinal design. With such a design we can conclude that sexuality education was related to an increase in the willingness to intervene when witnessing LGBTQ name-calling and a decrease in name-calling. However, we should consider that those schools with more comprehensive sexuality education may have been more positive toward gender and sexual diversity than the schools that did not offer comprehensive sexuality education. To tease apart these different trajectories, a longitudinal study with a measurement of sexuality education as perceived by students and teachers, and reported by principals, in addition to a baseline measurement would be advised, as well as research using randomized control trials to test causal mechanisms.

### Implications of the Current Study

Although studies have shown that males are important contributors to homophobic behavior in schools, and the persistence of homophobic masculinity norms (Poteat et al. 2011), many of the (preventive) interventions aimed at improving school climate do not address these issues directly. Our findings suggest that some males do intervene when they witness LGBTQ name-calling, but they are not as likely to do so as females. However, the current study also shows that only males’, and not females’, tendency to intervene when they witness LGBTQ name-calling increased in relation to receiving extensive sexuality education. Therefore, ways to increase defender behavior and improve school climate for, and by, males are crucial to consider in the design of (preventive) interventions. Moreover, it is important to consider differences in attitudes toward different gender and sexual identities, and the required efforts to decrease biases among males *and* females (Worthen 2013).

## Conclusions

The current study confirms that comprehensive sexuality education is related to an improvement of school climate over time. For school administrators, counselors, *and* teachers, the findings reiterate the importance of the curricula that they offer. Although in the current study teachers and students are reported to intervene more often in response to LGBTQ name-calling than in U.S. samples (Kosciw et al. 2016), the percentage that “never” intervenes (22.4% of teachers and 50.5% of fellow students) underlines the responsibility to educate students and model accepting behaviors. Thus, empowering youth *and* school personnel to create a more inclusive school climate may be the missing link in improving the school environment and health of LGBTQ students.
